# Prevalence, Determinants, and Clinical Associations of High-Sensitivity Cardiac Troponin in Patients Attending Emergency Departments

**DOI:** 10.1016/j.amjmed.2018.10.002

**Published:** 2019-01

**Authors:** Kuan Ken Lee, Ala Noaman, Amar Vaswani, Matthew Gibbins, Megan Griffiths, Andrew R. Chapman, Fiona Strachan, Atul Anand, David A. McAllister, David E. Newby, Alasdair J. Gray, Nicholas L. Mills, Anoop S.V. Shah

**Affiliations:** a BHF Centre for Cardiovascular Science, University of Edinburgh, UK; b Institute of Health and Wellbeing, University of Glasgow, UK; c Department of Emergency Medicine, Royal Infirmary of Edinburgh, UK; d Emergency Medicine Research Group Edinburgh (EMERGE), UK

**Keywords:** Acute coronary syndrome, Diagnosis, High-sensitivity cardiac troponin, Myocardial infarction, Myocardial injury

## Abstract

**Background:**

High-sensitivity cardiac troponin assays may improve the diagnosis of myocardial infarction but increase the detection of elevated cardiac troponin in patients without acute coronary syndrome.

**Methods:**

In a prospective cohort study, we evaluated the prevalence, determinants, and outcome of patients with elevated cardiac troponin attending the emergency department without suspected acute coronary syndrome. We measured high-sensitivity cardiac troponin in 918 consecutive patients attending the emergency department without suspected acute coronary syndrome who had blood sampling performed by the attending clinician. Elevated high-sensitivity cardiac troponin I was defined as concentrations above the sex-specific 99th percentile threshold. Clinical demographics, physiological measures, and all-cause mortality at 1 year associated with elevated high-sensitivity cardiac troponin concentrations were recorded.

**Results:**

Elevated cardiac troponin concentration occurred in 114 (12.4%) patients, of whom 2 (0.2%), 3 (0.3%), and 109 (11.9%) were adjudicated as type 1 myocardial infarction, type 2 myocardial infarction, and myocardial injury, respectively. Elevated troponin concentrations were associated with increasing age, worsening renal function, multimorbidity, and adverse physiology. Across a total of 912 patient-years follow-up, cardiac troponin concentration was a strong predictor of death (hazard ratio [HR] 1.26 per 2-fold increase, 95% confidence interval [CI] 1.06 to 1.49) independent of age, sex, multimorbidity, and adverse physiology.

**Conclusions:**

High-sensitivity cardiac troponin concentrations were elevated in 1 in 8 consecutive patients without suspected acute coronary syndrome attending the emergency department and were associated with increasing age, multimorbidity, adverse physiology, and death. Elevated cardiac troponin in unselected patients predominantly reflects myocardial injury rather than myocardial infarction.

**Clinical Significance**

• Elevated high-sensitivity cardiac troponin concentration is common in patients presenting to the emergency department without suspected acute coronary syndrome.
• The vast majority of these elevated cardiac troponin concentration reflects myocardial injury rather than myocardial infarction.
• Cardiac troponin concentration is strongly associated with age, comorbidity, adverse physiology at presentation, and worse outcomes.

Alt-text: Unlabelled box

## Background

Although cardiac troponin is integral to the diagnosis of myocardial infarction,^1^ the approach to testing varies markedly across different healthcare systems.^2^, ^3^, ^4^ In some centers, cardiac troponin is used as a screening test, irrespective of the clinical presentation.^4^ In a representative sample of patients attending emergency departments in the United States, cardiac troponin was measured in 1 in 5 patients.^5^ Moreover, in more than half of those who were subsequently hospitalized, one-third of patients did not have any cardiac symptoms.^5^

We recently assessed the variation in patient selection for high-sensitivity troponin testing between emergency departments and its impact on the diagnosis of type 1 myocardial infarction.^6^ Compared to the United Kingdom, cardiac troponin testing was performed more widely in the United States, resulting in a much lower prevalence (4.2% vs 14.5%) and positive predictive value (16.4% vs 59.7%) for type 1 myocardial infarction in those patients tested.^6^ These findings highlight the importance of patient selection to optimize the diagnostic utility of cardiac troponin testing for type 1 myocardial infarction.^6^

High-sensitivity cardiac troponin assays have been widely implemented in many countries and have improved the diagnosis of myocardial infarction^1^, ^7^ and risk stratification in patients with suspected acute coronary syndrome.^7^ However, the high-sensitivity test also increases the detection of elevated cardiac troponin in many cardiac and noncardiac conditions that are unrelated to acute coronary syndrome.^8^, ^9^, ^10^ As such, the introduction of a high-sensitivity cardiac troponin assay may contribute to diagnostic uncertainty, particularly in healthcare settings where indiscriminate testing is high.^2^, ^5^

Here, we aim to evaluate the prevalence of elevated high-sensitivity cardiac troponin concentrations in consecutive patients without suspected acute coronary syndrome in emergency department and describe the determinants and clinical associations of high-sensitivity cardiac troponin concentrations.

## Methods

### Study Population

We prospectively identified consecutive patients presenting to the Emergency Department at the Royal Infirmary of Edinburgh, Scotland, between July 5 and 16, 2013. The Royal Infirmary of Edinburgh Emergency Department serves a population of more than 1 million across the south east of Scotland. It is an adult tertiary emergency department, serving as regional referral center for trauma, surgery, and cardiology, assessing approximately 120,000 patients annually.

**Supplementary Figure 1:**
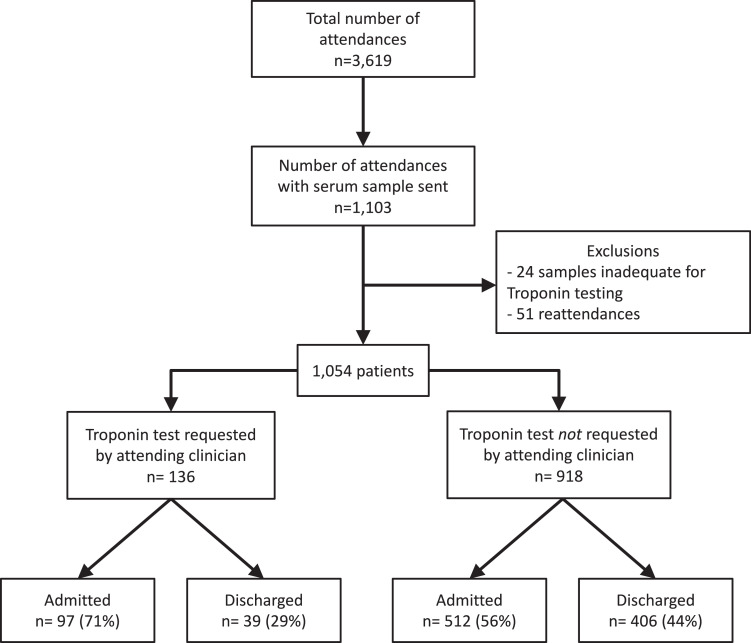
Flow diagram of the study population of unselected patients attending the Emergency Department

All patients without suspected acute coronary syndrome in whom the attending clinician performed blood sampling at presentation and did not request a cardiac troponin test were eligible for inclusion in this analysis (Supplementary Figure 1). Excess serum from blood samples were used to measure high-sensitivity cardiac troponin I. These results were not reported to the attending clinician to guide clinical care. Patients who had a previous admission during the study period, or in whom there was insufficient sample volume for analysis, were excluded. This study was performed with approval from the National Health Service Research Scotland BioResource and Tissue Governance Unit in accordance with the Declaration of Helsinki. As surplus material was acellular, individual consent from each patient was not sought. However, to comply with our governance policy, we used an encrypted identification number for each individual troponin result measured on excess serum to ensure that they were never linked to patient identifiers. We have employed a similar approach for previous clinical trials and cohort studies conducted in this manner.^6^, ^7^, ^8^^,^^11^, ^12^

### Troponin Assay

Excess serum was analyzed using the ARCHITECT*_STAT_* high-sensitive troponin I assay (Abbott Laboratories, Abbott Park, IL, USA). This assay has a limit of detection of 1.2 ng/L and the inter-assay CV <10% at 4.7 ng/L. The 99th percentile upper reference limit (URL) is 34 ng/L in men and 16 ng/L in women.^12^, ^13^

### Clinical Characteristics

Baseline clinical characteristics and investigations were obtained from a standardized electronic patient record (TrakCare, InterSystems Corporation, Cambridge, MA, USA) as previously described.^8^, ^12^^,^^14^ These included medical history, cardiovascular risk factors, presenting symptoms, medication use, and electrocardiographic abnormalities. Hyperlipidemia and hypertension were defined as a documented history of the condition or by the respective use of lipid-lowering or antihypertensive medications. The National Early Warning Score (NEWS) was calculated for patients incorporating six simple physiological variables (ie pulse rate, blood pressure, temperature, oxygen saturations, level of consciousness, and respiratory rate measured at admission; Supplementary Table 1).^15^

### Classification of Myocardial Injury and Myocardial Infarction

Elevated cardiac troponin concentration was defined using sex-specific thresholds at the 99th percentile URL.^1^, ^7^^,^^16^ Two cardiologists reviewed all clinical information independently, including noninvasive and invasive investigations and outcomes from admission to 30 days as described previously.^7^, ^11^^,^^12^ Patients with elevated cardiac troponin were classified according to the universal definition of myocardial infarction.^7^, ^12^ Type 1 myocardial infarction was defined in which myocardial necrosis occurred in the context of a presentation with symptoms suggestive of an acute coronary syndrome or evidence of myocardial ischemia on the electrocardiogram. Patients with myocardial necrosis and symptoms or signs of myocardial ischemia as a result of increased oxygen demand or decreased supply (eg tachyarrhythmia, hypotension, or anemia) due to an alternative diagnosis were classified as type 2 myocardial infarction. Myocardial injury was defined as elevated cardiac troponin concentration without signs or symptoms of myocardial ischemia. A third adjudicator resolved any discrepancies in adjudication through in-depth review of source documents.

### Clinical Outcomes

Death from any cause was identified from regional and national registries including the General Register of Scotland,^17^ allowing capture of all deaths in the hospital and in the community, ensuring complete follow-up in all patients. Patients who were not residents of Scotland were censored at the time of discharge.

### Statistical Analysis

**Supplementary Figure 2:**
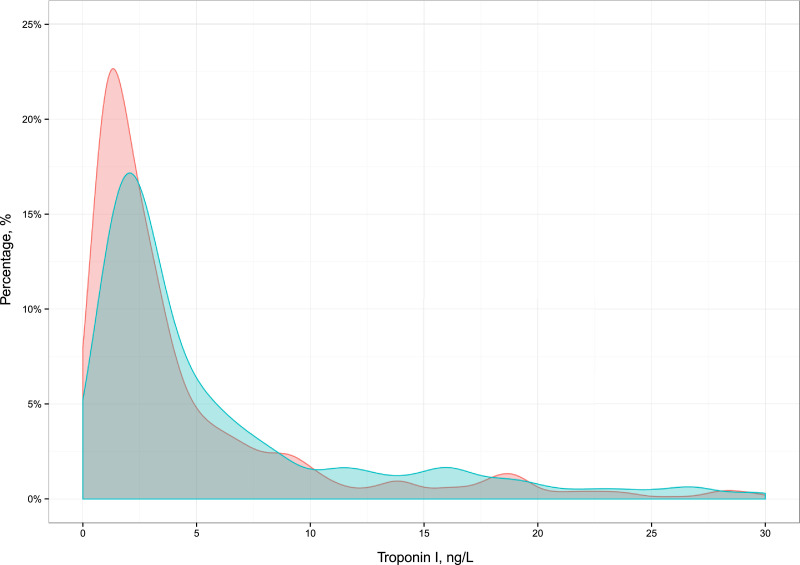
Density plot for the distribution of cardiac troponin I concentration in unselected patients attending the Emergency Department stratified by sex with blue as males and red as females.

Patients were divided into four groups a priori: those patients with cardiac troponin concentrations >99th percentile URL (men >34 ng/L and women >16 ng/L; group 4) and the remaining patients separated into three equal tertiles (groups 1-3). The distribution of cardiac troponin differed in men and women (Supplementary Figure 2), and therefore, they were grouped separately. Summary statistics were derived for all patients stratified by these four groups. Continuous variables were compared using parametric and nonparametric tests as appropriate.

We calculated the prevalence of elevated cardiac troponin across the whole population and stratified by age, sex, known ischemic heart disease, renal function, and comorbidities (eg hypertension, ischemic heart disease, peripheral vascular disease, diabetes mellitus, and cerebrovascular disease). Where the prevalence was close to 0%, we calculated the 95% confidence interval (CI) using a Bayesian approach by sampling from a binomial likelihood with a noninformative Jeffreys prior (ß-distribution shape parameters both equal to 0.5).

Factors associated with elevated cardiac troponin concentrations were modeled using logistic regression with adjustment for age, sex, renal function, multimorbidity (eg, hypertension, diabetes mellitus, ischemic heart disease, cerebrovascular disease, or peripheral vascular disease) and NEWS. Occurrence of death was modelled using a Cox-proportional hazards regression with troponin (natural log) as the explanatory variable both as a single variable and after adjusting for age, sex, renal function, and NEWS. We examined the linearity of the association between troponin concentration and risk of death using Cox-proportional regression models with smoothing splines for high-sensitivity cardiac troponin concentrations and 4 degrees of freedom. Cox-regression models were constructed and evaluated for the proportional hazards assumption. Analyses were performed in R Version 3.5.1 Statistical significance was taken as a two-sided P < 0.05.

## Results

### Study Population

There were 3619 attendances to the Emergency Department across the study period, and blood samples were obtained as part of routine clinical care in 1103 (Supplementary Figure 1). Prior inclusion in the study or insufficient blood sample for analysis excluded 49 patients. The attending clinician requested cardiac troponin in 12.9% (n=136) of all patients who had blood drawn. Patients who had a troponin test requested were more likely to have presented to the emergency department with chest pain (79.4% vs 8.3%, P-value <0.001), have cardiac risk factors, and past medical history of ischemic heart disease (Supplementary Table 2). These patients (n = 136) were excluded, resulting in a final study population of 918 patients (Supplementary Figure 1).

### Prevalence of Elevated Cardiac Troponin Concentration

**Figure 1:**
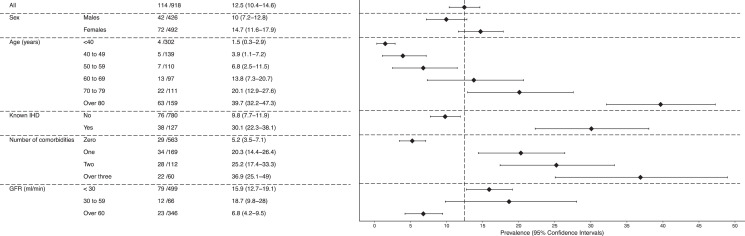
Prevalence of elevated cardiac troponin concentration in the emergency department stratified by gender, age, comorbidity, and renal function in those patients in whom cardiac troponin was not requested by the attending clinician.

The prevalence of elevated cardiac troponin was 12.4% (n=114/918). Two (0.2%) and 3 (0.3%) patients with elevated cardiac troponin concentrations were adjudicated as having had type 1 and type 2 myocardial infarction, respectively, with the remaining 109 patients adjudicated as having myocardial injury (11.9%) (Figure 1, Supplementary Table 3).

### Determinants of Elevated Cardiac Troponin Concentration

Patients with elevated cardiac troponin (group 4) were older (78 ± 17 vs 34 ± 13 years, P < 0.001) and more likely to have comorbid conditions than those with the lowest cardiac troponin concentrations (group 1) (Table 1). Patients with elevated cardiac troponin were also more likely to have adverse physiology (medium or high-risk NEWS, 19.5% vs 2.4%, P<0.001), lower hemoglobin concentrations (12.1±2.4 vs 13.7±1.6 g/dL, P<0.001), and higher creatinine concentrations (1.30±1.00 vs 0.80±0.16 mg/dL).

**Table 1:** Baseline Characteristics of Patients Stratified by High-Sensitivity Cardiac Troponin Concentration

	All *(n = 918)*	Group 1 *(n = 316)*	Group 2 *(n = 236)*	Group 3 *(n = 252)*	Group 4 *(n = 114)*	P-value
*Range of troponin concentration, ng/L*						
Males		≤3	3 to 6	6 to 34	>34	
Females		≤1	2 to 4	4 to 16	>16	
Male sex	426 (46.4)	156 (49.4)	101 (42.8)	127 (50.4)	42 (36.8)	0.043
Age (years)	53.0 ± 23.0	34.0 ± 13.1	50.5 ± 17.9	67.9 ± 18.2	77.6 ± 16.5	<0.001
Chest pain	75 (8.3)	24 (7.7)	18 (7.7)	21 (8.4)	12 (10.7)	0.776
*Risk factors*						
Smoker	258 (33.2)	102 (37.6)	73 (36.5)	58 (27.0)	25 (27.8)	0.04
Ex-smoker	119 (15.3)	11 (4.1)	21 (10.5)	61 (28.2)	26 (28.9)	<0.001
Hypertension	257 (28.4)	16 (5.2)	54 (23.1)	116 (46.6)	71 (62.8)	<0.001
Hyperlipidemia	230 (25.4)	16 (5.2)	49 (20.9)	109 (43.8)	56 (49.1)	<0.001
Family history	9 (1.0)	1 (0.3)	3 (1.3)	3 (1.2)	2 (1.8)	0.491
*Past medical history*						
Ischemic heart disease	127 (14.0)	9 (2.9)	18 (7.7)	62 (24.9)	38 (33.3)	<0.001
Myocardial infarction	61 (6.7)	5 (1.6)	7 (3.0)	31 (12.4)	18 (15.8)	<0.001
Cerebrovascular disease	83 (9.2)	3 (1.0)	17 (7.3)	37 (14.9)	26 (22.8)	<0.001
Peripheral vascular disease	43 (4.7)	4 (1.3)	5 (2.1)	19 (7.6)	15 (13.2)	<0.001
Diabetes	81 (8.9)	13 (4.2)	18 (7.7)	35 (13.9)	15 (13.2)	<0.001
*Previous revascularization*						
PCI	28 (3.1)	4 (1.3)	5 (2.1)	13 (5.2)	6 (5.3)	0.022
CABG	19 (2.1)	0 (0.0)	1 (0.4)	9 (3.6)	9 (7.9)	<0.001
*Admission physiological parameters*						
Medium or high risk NEWS (≥5)	34 (5.7)	5 (2.4)	4 (2.7)	9 (5.5)	16 (19.5)	<0.001
Respiratory rate (bpm)	18.3 ± 4.3	17.6 ± 3.7	17.3 ± 3.0	18.5 ± 3.8	21.4 ± 6.5	<0.001
Temperature (°C)	36.9 ± 0.9	36.8 ± 0.8	36.8 ± 0.8	37.0 ± 0.9	37.1 ± 1.2	0.013
Pulse rate (bpm)	87.7 ± 22.1	85.8 ± 19.0	86.1 ± 20.4	87.6 ± 22.7	95.9 ± 28.8	0.001
Systolic BP (mm Hg)	130.5 ± 22.3	127.0 ± 16.7	130.4 ± 21.8	134.9 ± 25.5	130.1 ± 26.8	0.003
Oxygen saturations (%)	96.8 ± 3.0	97.9 ± 1.8	97.1 ± 2.1	96.0 ± 3.3	95.1 ± 4.7	<0.001
On oxygen	52 (6.3)	4 (1.5)	6 (2.8)	19 (8.3)	23 (21.9)	<0.001
Consciousness						0.01
Alert	880 (96.6)	306 (98.1)	227 (97.0)	243 (96.8)	104 (91.2)	
Verbal	14 (1.5)	3 (1.0)	5 (2.1)	1 (0.4)	5 (4.4)	
Pain	7 (0.8)	2 (0.6)	0 (0.0)	4 (1.6)	1 (0.9)	
Unresponsive	10 (1.1)	1 (0.3)	2 (0.9)	3 (1.2)	4 (3.5)	
Killip class						<0.001
0	6 (0.7)	1 (0.3)	0 (0.0)	2 (0.8)	3 (2.7)	
1	819 (90.8)	304 (99.0)	222 (95.7)	212 (84.8)	81 (71.7)	
2	65 (7.2)	2 (0.7)	9 (3.9)	30 (12.0)	24 (21.2)	
3	11 (1.2)	0 (0.0)	1 (0.4)	6 (2.4)	4 (3.5)	
4	1 (0.1)	0 (0.0)	0 (0.0)	0 (0.0)	1 (0.9)	
*Hematology and biochemistry*						
Hemoglobin (g/dL)	13.3 ± 2.0	13.9 ± 1.5	13.5 ± 1.7	12.9 ± 2.1	12.1 ± 2.4	<0.001
Creatinine (mg/dL)	0.96 ± 0.74	0.80 ± 0.16	0.88 ± 0.78	1.07 ± 0.90	1.30 ± 1.00	<0.001
Urea (mg/dL)	5.9 ± 3.7	4.4 ± 1.5	5.1 ± 2.4	7.0 ± 4.1	9.7 ± 5.5	<0.001
*Admission drugs*						
Aspirin	130 (14.5)	7 (2.3)	19 (8.2)	66 (27.0)	38 (33.9)	<0.001
Clopidogrel	62 (6.9)	5 (1.6)	10 (4.3)	33 (13.5)	14 (12.5)	<0.001
Beta-blockers	109 (12.1)	9 (2.9)	24 (10.3)	45 (18.4)	31 (27.7)	<0.001
ACE-I/ARB	143 (15.9)	9 (2.9)	34 (14.7)	63 (25.8)	37 (33.0)	<0.001
Statin	192 (21.4)	13 (4.2)	43 (18.5)	91 (37.3)	45 (40.2)	<0.001
Warfarin	35 (3.9)	1 (0.3)	7 (3.0)	16 (6.6)	11 (9.9)	<0.001
PPI	184 (20.5)	28 (9.0)	52 (22.4)	65 (26.6)	39 (34.8)	<0.001
*Admission ECG*						
ST elevation	5 (1.0)	1 (0.6)	2 (1.7)	0 (0.0)	2 (2.3)	0.261
ST depression	9 (1.7)	0 (0.0)	1 (0.8)	2 (1.2)	6 (6.9)	0.001
T-wave inversion	34 (6.5)	3 (2.0)	6 (5.0)	16 (10.0)	9 (10.3)	0.012
New LBBB	5 (1.0)	0 (0.0)	0 (0.0)	1 (0.6)	4 (4.6)	0.002
Old LBBB	13 (2.5)	0 (0.0)	0 (0.0)	8 (5.0)	5 (5.7)	0.002
RBBB	15 (2.9)	2 (1.3)	1 (0.8)	7 (4.4)	5 (5.7)	0.073
*Outcomes*						
Admitted to hospital	512 (56.2)	128 (41.0)	119 (50.6)	169 (67.6)	96 (84.2)	<0.001
30-day death	32 (3.5)	2 (0.6)	1 (0.4)	7 (2.8)	22 (19.3)	<0.001
1-year death	102 (11.1)	6 (1.9)	13 (5.5)	40 (15.9)	43 (37.7)	<0.001

Values are n (%) or mean ± SD.

ACE-I/ARB = angiotensin converting enzyme inhibitor/angiotensin receptor blocker; BP = blood pressure; bpm = breaths/beats per minute; CABG = coronary artery bypass grafting; ECG = electrocardiogram; LBBB = left bundle branch block; NEWS = National Early Warning Score; PCI = percutaneous coronary intervention; PPI = proton pump inhibitor; RBBB = right bundle branch block.

On logistic regression modeling, both age (model 1: odds ratio [OR] 2.13, 95% CI 1.78 to 2.62 per 10-year increment) and comorbidity (model 2: OR 10.11, 95% CI 4.3 to 23.81 for 3 or more comorbidities compared to none) were strongly associated with elevated cardiac troponin after adjusting for sex, renal function, and NEWS (Table 2). When adjusting for both age and comorbidity, age remained a powerful determinant of elevated cardiac troponin (model 3: OR 2.07, 95% CI 1.70 to 2.59 per 10 years increment, P<0.001).

**Table 2:** Logistic Regression Models for Determinants of Elevated Cardiac Troponin in Unselected Patients Attending the Emergency Department

Variable	Model 1, OR (95% CI)	Model 2, OR (95% CI)	Model 3, OR (95% CI)
Age, per 10 years	2.13 (1.78 – 2.62)*	-	2.07 (1.70 – 2.58)*
Creatinine, per 0.11 mg/dL	1.03 (1.00 – 1.07)†	1.03 (1.00 – 1.06)	1.03 (1.00 – 1.06)†
Sex (Male)	0.56 (0.31 – 1.01)	0.38 (0.21 – 0.67)*	0.52 (0.27 – 0.94)†
NEWS, per 1 unit	1.55 (1.33 – 1.83)*	1.57 (1.36 – 1.83)*	1.56 (1.33 – 1.84)*
Comorbidity			
None (referent)	—	1	1
1	—	4.59 (2.37 – 8.99)*	1.24 (0.58 – 2.63)
2	—	4.49 (2.07 – 9.62)*	0.95 (0.40 – 2.25)
Over 3	—	10.11 (4.3 – 23.81)*	2.16 (0.85 – 5.47)
AIC (model fit)	325	386	324

AIC =Akaike Information Criterion; CI, confidence interval; NEWS = National Early Warning Score; OR = odds ratio.

⁎ p value <0.001.

† p value <0.05.

**Supplementary Table 1:** Parameters and Scoring System of the National Early Warning Score (NEWS)

Points	3	2	1	0	1	2	3
Physiological parameter							
Respiratory rate, breaths per minute	≤8	–	9 to 11	12 to 20	–	21 to 24	≥25
Oxygen saturations, %	≤91	92 to 93	94 to 95	≥96	–	–	
Any supplemental oxygen	–	Yes	–	No	–	–	–
Temperature, ^o^C	≤35.0		35.1 to 36.0	36.1 to 38.0	38.1 to 39	≥39.1	–
Systolic blood pressure, mmHg	≤90	91 to 100	101 to 110	111 to 219	–	–	–
Heart rate, beats per minute	≤40	–	41 to 50	51 to 90	91 to 110	111 to 130	≥131
Consciousness	–	–	–	Alert	–	Voice, Pain or Unresponsive	–

**Supplementary Table 2:** Baseline Characteristics of Consecutive Patients in the Emergency Department With and Without Cardiac Troponin Testing Requested by the Attending Clinician

		Troponin requested by clinician		
	All *(n=1,054)*	No *(n=918)*	Yes *(n=136)*	P-value
Male sex	505 (47.9)	426 (46.4)	79 (58.1)	0.014
Age (years)	54.5 ± 22.6	53.0 ± 23.0	64.9 ± 16.4	<0.001
Elevated cardiac troponin*	144 (13.7)	114 (12.4)	30 (22.1)	<0.001
*Presenting complaint*				
Chest pain, n (%)	183 (17.6)	75 (8.3)	108 (79.4)	<0.001
Abdominal pain, n (%)	160 (15.2)	156 (17.0)	4 (2.9)	<0.001
Fall/collapse, n (%)	158 (15.0)	151 (16.4)	7 (5.1)	0.001
Dyspnea, n (%)	57 (5.4)	51 (5.6)	6 (4.4)	0.728
Confusion, n (%)	18 (1.7)	17 (1.9)	1 (0.7)	0.56
Dizziness, n (%)	14 (1.3)	13 (1.4)	1 (0.7)	0.806
Other, n (%)	464 (44.0)	455 (49.6)	9 (6.6)	<0.001
*Risk factors*				
Smoker	299 (33.1)	258 (33.2)	41 (32.5)	0.957
Ex-smoker	154 (17.1)	119 (15.3)	35 (27.8)	0.001
Hypertension	337 (32.4)	257 (28.4)	80 (59.3)	<0.001
Hyperlipidemia	299 (28.7)	230 (25.4)	69 (51.5)	<0.001
Family history	26 (2.5)	9 (1.0)	17 (12.7)	<0.001
*Past medical history*				
Ischemic heart disease	193 (18.5)	127 (14.0)	66 (48.9)	<0.001
Myocardial infarction	109 (10.5)	61 (6.7)	48 (35.6)	<0.001
Cerebrovascular disease	99 (9.5)	83 (9.2)	16 (11.9)	0.403
Peripheral vascular disease	50 (4.8)	43 (4.7)	7 (5.2)	0.992
Diabetes mellitus	106 (10.1)	81 (8.9)	25 (18.5)	0.001
Previous revascularisation				
PCI	52 (5.0)	28 (3.1)	24 (17.8)	<0.001
CABG	32 (3.1)	19 (2.1)	13 (9.6)	<0.001
*Admission physiological parameters*				
Respiratory rate (bpm)	18.2 ± 4.2	18.3 ± 4.3	17.8 ± 3.4	0.253
Temperature (^o^C)	36.9 ± 0.9	36.9 ± 0.9	36.6 ± 0.6	0.003
Pulse rate (bpm)	86.9 ± 22.3	87.7 ± 22.1	81.8 ± 23.0	0.008
Systolic BP (mmHg)	130.5 ± 22.2	130.5 ± 22.3	130.8 ± 21.9	0.887
Oxygen saturations (%)	96.8 ± 2.9	96.8 ± 3.0	96.7 ± 2.2	0.7
On oxygen	62 (6.6)	52 (6.3)	10 (8.1)	0.598
Consciousness				0.515
Alert	1014 (96.8)	880 (96.6)	134 (98.5)	
Verbal	14 (1.3)	14 (1.5)	0 (0.0)	
Pain	8 (0.8)	7 (0.8)	1 (0.7)	
Unresponsive	11 (1.1)	10 (1.1)	1 (0.7)	
Killip class				0.012
0	6 (0.6)	6 (0.7)	0 (0.0)	
1	930 (89.7)	819 (90.8)	111 (82.2)	
2	85 (8.2)	65 (7.2)	20 (14.8)	
3	15 (1.4)	11 (1.2)	4 (3.0)	
4	1 (0.1)	1 (0.1)	0 (0.0)	
*Hematology and biochemistry*				
Hemoglobin (g/dL)	13.3 ± 1.9	13.3 ± 2.0	13.2 ± 1.7	0.590
Creatinine (mg/dL)	0.97 ± 0.72	0.96 ± 0.74	1.04 ± 0.58	0.207
Urea (mg/dL)	6.1 ± 3.7	5.9 ± 3.7	6.8 ± 3.8	0.017
Troponin I (ng/L)	3.0 [2.0, 10.0]	3.0 [2.0, 9.0]	5.0 [3.0, 19.5]	<0.001
*Admission drugs*				
Aspirin	180 (17.5)	130 (14.5)	50 (37.6)	<0.001
Clopidogrel	81 (7.9)	62 (6.9)	19 (14.3)	0.005
Beta-blockers	149 (14.5)	109 (12.1)	40 (30.1)	<0.001
ACE-I/ARB	189 (18.3)	143 (15.9)	46 (34.6)	<0.001
Statin	249 (24.2)	192 (21.4)	57 (42.9)	<0.001
Warfarin	44 (4.3)	35 (3.9)	9 (6.8)	0.195
PPI	233 (22.6)	184 (20.5)	49 (36.8)	<0.001
*Admission electrocardiogram*				
ST elevation	13 (2.0)	5 (1.0)	8 (6.0)	0.001
ST depression	21 (3.2)	9 (1.7)	12 (9.0)	<0.001
T-wave inversion	58 (8.9)	34 (6.5)	24 (18.0)	<0.001
New LBBB	11 (1.7)	5 (1.0)	6 (4.5)	0.014
Old LBBB	17 (2.6)	13 (2.5)	4 (3.0)	0.982
RBBB	21 (3.2)	15 (2.9)	6 (4.5)	0.501
*Management*				
Chest pain nurse referral	36 (3.4)	5 (0.5)	31 (22.8)	<0.001
Cardiology referral	50 (4.8)	14 (1.5)	36 (26.5)	<0.001
Admitted to hospital	609 (58.2)	512 (56.2)	97 (71.3)	0.001
Echocardiogram	17 (1.6)	5 (0.5)	12 (8.8)	<0.001
Coronary angiogram	17 (1.6)	2 (0.2)	15 (11.0)	<0.001
PCI	10 (1.0)	0 (0.0)	10 (7.4)	<0.001
CABG	1 (0.1)	0 (0.0)	1 (0.7)	0.27
*Discharge drugs*				
Aspirin	192 (18.8)	131 (14.7)	61 (45.5)	<0.001
Clopidogrel	102 (10.0)	69 (7.8)	33 (24.6)	<0.001
Beta-blockers	152 (14.9)	106 (11.9)	46 (34.3)	<0.001
ACEI/ARB	181 (17.7)	134 (15.1)	47 (35.1)	<0.001
Statin	256 (25.0)	192 (21.5)	64 (47.8)	<0.001
Warfarin	44 (4.3)	33 (3.7)	11 (8.2)	0.03
Proton pump inhibitor	246 (24.0)	193 (21.7)	53 (39.6)	<0.001

Values are n (%), mean ± SD or median [IQR]

Abbreviations: bpm=breaths/beats per minute; BP=blood pressure; PCI= percutaneous coronary intervention, CABG=coronary artery bypass grafting, ACE-I/ARB=angiotensin converting enzyme inhibitor/ angiotensin receptor blocker, PPI=proton pump inhibitor

⁎ Consists of all patients with troponin levels above the 99^th^ percentile upper reference limit using sex specific diagnostic thresholds including those further classified as type 1 and type 2 myocardial infarction

**Supplementary Table 3:** Case Series of Patients Classified as Type 1 or Type 2 Myocardial Infarction in Whom the Attending Clinician Did Not Request Cardiac Troponin

Age	Sex	Classification	Presenting complaint	Known IHD	TnI, ng/L	Ischemia on ECG	Admitted	Clinical details
94	M	Type 1	Chest pain	No	1,377	Yes	Yes	Late presentation myocardial infarction, not for escalation of care, died in hospital
44	M	Type 1	Chest pain	No	132	No	No	Atypical history of chest pain
65	M	Type 2	Breathlessness	No	46	Yes	Yes	Patient with multiple comorbidities and admitted with shortness of breath secondary to exacerbation of COPD
94	F	Type 2	Chest pain	No	24	No	Yes	Admitted with fast ventricular response to atrial fibrillation and associated chest tightness
72	M	Type 2	Chest pain	No	36	Yes	Yes	Admitted with fast ventricular response to atrial fibrillation and associated chest pain with rate related left bundle branch block

**Supplementary Table 4:** Presenting Complaint in Patients Without Suspected Acute Coronary Syndrome and Cardiac Troponin Concentrations >99th Centile

Presenting complaint	Number *(n = 114)*	Percentage
Fall/Collapse	39	34.2%
Chest pain	11	9.6%
Dyspnea	10	8.8%
Abdominal pain	8	7.0%
Dizziness	4	3.5%
Confusion	3	2.6%
Other	39	34.2%

**Supplementary Table 5:** Primary Clinical Diagnostic Category of Patients with Cardiac Troponin Concentrations >99th Centile

Diagnostic category	Number *(n = 114)*	Percentage
Infective / sepsis	31	27%
Mechanical fall	15	13%
Other	14	12%
Cardiac*	12	11%
Respiratory†	11	10%
Trauma / Orthopaedic	10	9%
Gastrenterology / hepatology	7	6%
Renal	6	5%
Surgical	5	4%
Neurological	3	3%

⁎ In patients with a cardiac diagnosis, atrial fibrillation accounted for the majority (n=8).

† These refer to respiratory diagnoses other than pneumonia which was included within the Infective/sepsis category.

**Supplementary Table 6:** Primary Cause of Death for All Patients Without Suspected Acute Coronary Syndrome at 1 Year Follow-Up

Death category	Number *(n = 102)*	Percentage
Sepsis	20	19.6%
Cancer	12	11.8%
Respiratory	7	6.9%
Gastrointestinal	6	5.9%
Cardiac	5	4.9%
Stroke	3	2.9%
Vascular	3	2.9%
Renal	3	2.9%
Other	3	2.9%
Unknown	40	39.2%

### Outcomes of Patients with Elevated Cardiac Troponin Concentration

**Figure 2:**
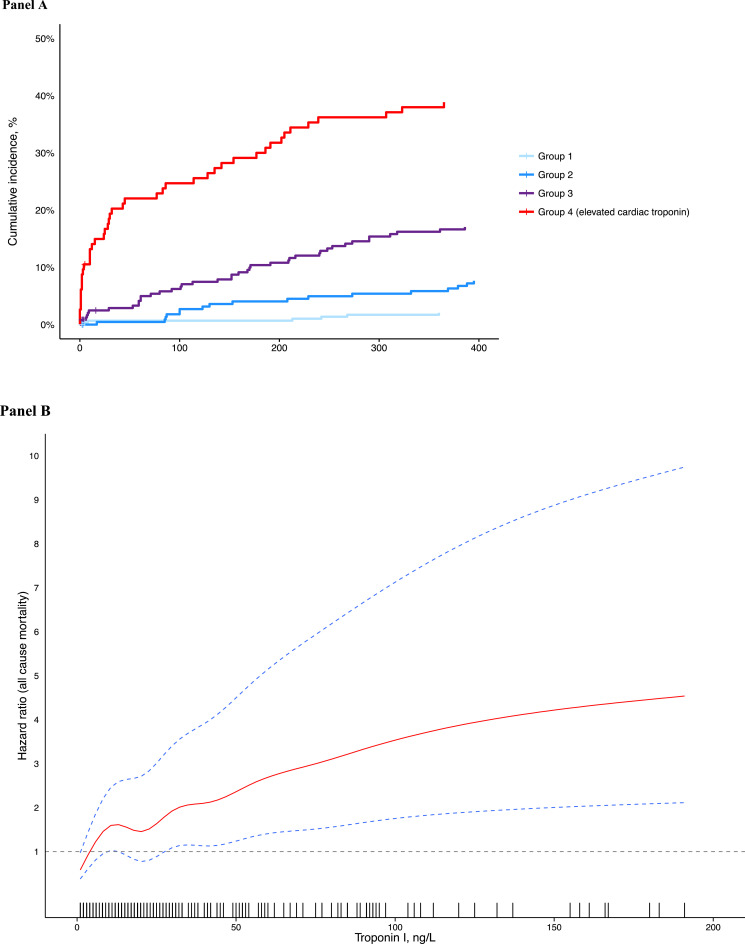
(**A**) Cumulative incidence for all-cause mortality in patients in whom cardiac troponin was not requested by the attending clinician, stratified by cardiac troponin concentration at presentation. (**B**) Association between cardiac troponin concentration and hazard ratio for death. Estimates obtained from a Cox regression model with penalized smoothing splines and adjusted for age, sex, and renal function. Rug plot shows density of data for given value of cardiac troponin.

Most patients with elevated cardiac troponin were admitted into hospital (96/114, 84.2%). Patients with elevated cardiac troponin (group 4) were more likely to have died at 30 days and 1 year than those with the lowest cardiac troponin concentrations (group 1) (19.3% vs 0.6% at 30 days, *P*-value (log-rank) <0.001 and 37.7% vs 1.9% at 1 year, *P*<0.001; Table 1 and Figure 2A). With time to event analysis, across a total of 912 patient-years of follow-up, cardiac troponin concentration was a strong predictor of death (hazard ratio [HR] 1.62, 95% CI 1.50 to 1.75, p<0.001 for every doubling of troponin concentration; Figure 2A and 2B). This association attenuated after adjusting for age, sex, multimorbidity, adverse physiology, and renal function (HR 1.26, 95% CI 1.06 to 1.49, *P*=0.003 for every doubling of troponin concentration). There was a near linear relationship between troponin concentration at presentation and risk of death (Figure 2B). The proportional hazards assumption for the Cox model was met (*P*=0.664).

## Discussion

Given the increasing use of high-sensitivity cardiac troponin assays in clinical practice and the potential diagnostic challenges that elevated troponin concentrations may pose, we evaluated the prevalence and factors associated with an elevated cardiac troponin concentration in consecutive patients to the Emergency Department who did not have suspected acute coronary syndrome in whom the attending clinician performed blood sampling. We make several important observations. First, in our center, approximately 1 in every 8 patients without suspected acute coronary syndrome who underwent blood sampling had an elevated cardiac troponin concentration. Second, age and multimorbidity were important factors associated with cardiac troponin concentration. Nearly half of all patients older than the age of 80 or with greater than 3 comorbid conditions had an elevated cardiac troponin concentration. Third, elevated cardiac troponin concentration was associated with adverse physiology at presentation. Age, serum creatinine, hemodynamic compromise, and hypoxia were strong predictors of an elevated cardiac troponin. Finally, most patients with elevated cardiac troponin were admitted into hospital, and every doubling in troponin concentration was associated with mortality independent of age, sex, renal function, and adverse physiology.

To our knowledge, this is the first study to evaluate the determinants of elevated cardiac troponin in consecutive patients Emergency Department patients without suspected acute coronary syndrome with several important strengths in the study design. We used a consecutive patient population attending the Emergency Department who underwent blood sampling to minimize selection bias. In comparison, previous studies have evaluated the frequency of type 2 myocardial infarction and myocardial injury only in patients who had troponin testing clinically requested.^10^, ^18^ We also used a clinically available high-sensitivity troponin I assay with excellent precision to measure cardiac troponin. This assay has superior analytical performance compared to previous generations of cardiac troponin assays with a limit of detection 10-fold lower than contemporary sensitive assays.^19^, ^20^ Finally, to ensure robust follow-up of patients, we used comprehensive local and national death registries.^8^, ^12^

In our study, elevated cardiac troponin concentration was common in patients without suspected acute coronary syndrome in whom troponin testing was not requested by the attending clinician. Age and admission physiology were the strongest independent factors associated with elevated troponin concentrations. Although elevated cardiac troponins are specific for indicating damage to the myocardium, they are not specific for the etiology of injury. As such, many acute and chronic pathological conditions are associated with elevated cardiac troponins.^20^, ^21^ Perhaps unsurprisingly, elevated cardiac troponin concentrations are common in a critical-care setting, and likely to be secondary to multiple mechanisms.^22^ Potential mechanisms of injury include direct toxicity, hemodynamic compromise, and, in some cases, increased thrombogenicity precipitating a type 1 myocardial infarction. Myocardial oxygen supply-demand imbalance is a major factor in the critically ill, and both hypotension and increased metabolic demand are correlated with troponin concentrations.^23^ In addition, elevated cardiac troponin are also associated with inflammatory cytokines that may cause direct myocardial toxicity.^24^, ^25^, ^26^, ^27^

So, what information do cardiac troponin levels convey in patients attending the emergency department? There are many cardiac and noncardiac conditions unrelated to type 1 myocardial infarction that may result in elevated troponin concentrations. The universal definition of myocardial infarction acknowledges this diversity by classifying elevated troponin levels in such instances as either type 2 myocardial infarction or myocardial injury.^16^ Our analysis confirms previous studies demonstrating close associations among troponin concentrations, acute illness severity, and increased risk of death.^8^, ^22^ These associations remain robust and independent of known important confounders including age, sex, multimorbidity, renal function, and adverse physiological measures.

Our study has several broad clinical implications when adopting high-sensitivity assays. First, the prevalence of elevated high-sensitivity cardiac troponin is high in patients attending the emergency department without suspected acute coronary syndrome. High-sensitivity assays were introduced into clinical practice in Australasia, Canada, and Europe in 2010, yet the first high-sensitivity assay has only recently received approval from the U.S. Food and Drug Administration for use in the United States.^20^, ^28^ Although lowering the diagnostic threshold of cardiac troponin using more sensitive assays does not result in an increased number of tests per se, it does result in an increased proportion of “positive” results and an increased detection of nonacute coronary syndrome pathologies.^2^, ^29^ Indiscriminate troponin testing in patients without signs or symptoms consistent with acute coronary syndrome should be discouraged because it is likely to increase the challenges involved in interpreting troponin test results for the attending clinician. Second, although troponin elevations detected with these novel assays identify patients at risk of death, there is currently no evidence that these patients with an elevated troponin who do not have a diagnosis of type 1 myocardial infarction would benefit from any additional therapy.^8^ Third, elevated troponin concentrations reflect cardiac damage analogous to creatinine for acute kidney injury or hypoxia in the context of acute lung injury. As such, elevated cardiac troponin concentrations not resulting from a primary coronary pathology (ie type 1 myocardial infarction) identify patients with an acute illness and adverse physiology. In our cohort, most patients with undisclosed elevated cardiac troponin concentration were admitted to hospital. Two patients (0.2%) were adjudicated as type 1 myocardial infarction of whom one patient was a 94-year old man diagnosed as a late presentation myocardial infarction clinically and received palliative care in the hospital, highlighting that the attending clinicians were good at discriminating those patients who required further in-patient investigation and care.

### Limitations

There were several limitations in our study. Our cohort originated from a single center. However, our Emergency Department is in a large tertiary center providing acute medical care to the southeast of Scotland. We would not therefore envisage our patient population to be different from other Emergency Departments and consider our results and interpretation generalizable. However, we acknowledge that this study was conducted during the summer, and therefore, the study population may not be representative of the patient population presenting to Emergency Departments during the winter months. We also did not have serial troponin concentrations and are not able to comment on the whether there was an acute rise or fall in troponin concentrations. As such, our classification of the myocardial injury is based on a single baseline sample and may have underestimated the prevalence of myocardial infarction as a result of misclassification. Moreover, we only measured troponin concentration in patients who had blood sampling performed by the attending clinician. Although this might have introduced some confounding by indication, we believe this ensured that our study is representative of the broad population of patients presenting to the Emergency Department who were sufficiently unwell to require blood testing. It is in this group of patients in which diagnostic uncertainty may arise should troponin testing is requested indiscriminately.

## Conclusion

This is the first study that has evaluated high-sensitivity cardiac troponin concentrations in consecutive patients attending an Emergency Department without suspected acute coronary syndrome, in whom blood sampling was undertaken by the attending physician. Our analysis shows that elevated cardiac troponin concentrations are common in patients without suspected acute coronary syndrome. Troponin concentration is strongly associated with age, comorbidities, adverse physiology at presentation, and subsequent poorer outcomes. As high-sensitivity cardiac troponin assays are adopted more widely, an improved understanding of the prevalence, determinants, and clinical associations of cardiac troponin in patients without acute coronary syndrome is required to ensure that test results are interpreted correctly and the subsequent clinical management is appropriate.
